# The influence of transmitted and non-transmitted parental BMI-associated alleles on the risk of overweight in childhood

**DOI:** 10.1038/s41598-020-61719-3

**Published:** 2020-03-16

**Authors:** Theresia M. Schnurr, Camilla S. Morgen, Dmitrii Borisevich, Robin N. Beaumont, Line Engelbrechtsen, Lars Ängquist, Christian T. Have, Rachel M. Freathy, George Davey Smith, Ellen A. Nohr, Torben Hansen, Thorkild I. A. Sørensen

**Affiliations:** 1 Novo Nordisk Foundation Center for Basic Metabolic Research, Faculty of Health and Medical Sciences, University of Copenhagen, Copenhagen, Denmark; 2 National Institute of Public Health, University of Southern Denmark, Copenhagen, Denmark; 3 Genetics of Complex Traits, University of Exeter Medical School, University of Exeter, Royal Devon & Exeter Hospital, Exeter, UK; 4 Department of Gynecology and Obstetrics, Herlev Hospital, Herlev, Denmark; 5 MRC Integrative Epidemiology Unit at the University of Bristol, Bristol, UK; 6 Research Unit for Gynaecology and Obstetrics, Department of Clinical Research, University of Southern Denmark, Odense, Denmark; 7 Section of Epidemiology, Department of Public Health, Faculty of Health and Medical Sciences, University of Copenhagen, Copenhagen, Denmark

**Keywords:** Genetics research, Paediatric research

## Abstract

Overweight in children is strongly associated with parental body mass index (BMI) and overweight. We assessed parental transmitted and non-transmitted genetic contributions to overweight in children from the Danish National Birth Cohort by constructing genetic risk scores (GRSs) from 941 common genetic variants associated with adult BMI and estimating associations of transmitted maternal/paternal and non-transmitted maternal GRS with child overweight. Maternal and paternal BMI (standard deviation (SD) units) had a strong association with childhood overweight [Odds ratio (OR): 2.01 (95% confidence interval (CI) 1.74; 2.34) and 1.64 (95% CI 1.43; 1.89)]. Maternal and paternal transmitted GRSs (SD-units) increased odds for child overweight equally [OR: 1.30 (95% CI 1.16; 1.46) and 1.30 (95% CI 1.16; 1.47)]. However, both the parental phenotypic and the GRS associations may depend on maternal BMI, being weaker among mothers with overweight. Maternal non-transmitted GRS was not associated with child overweight [OR 0.98 (95% CI 0.88; 1.10)] suggesting no specific influence of maternal adiposity as such. In conclusion, parental transmitted GRSs, based on adult BMI, contribute to child overweight, but in overweight mothers other genetic and environmental factors may play a greater role.

## Introduction

Parental overweight is a potent risk factor for childhood overweight^1^ and both maternal and paternal body mass index (BMI) are associated with offspring BMI^2,3^. While genetic factors in both parents, transmitted to the children, may explain a major part of these phenotypic associations, shared environmental factors may also operate, as long as they are living together^4–8^. Greater maternal adiposity may enhance the risk of overweight in their children, independent of genetic transmission to the child, by altering the environment before, during or after the pregnancy (we will subsequently refer to those as specific maternal effects)^2,9^. Evidence of the specific maternal effects have been reported by a number of studies, which compared the strength of the associations of maternal and paternal BMI with childhood BMI^2,10^, whereas other studies suggested little or no such effects^3,11,12^. Two studies using intergenerational Mendelian randomization methods, in which maternal genetic variants associated with BMI were used as instrumental variables for greater maternal adiposity, did not find support for effects of the non-transmitted variants on child BMI^13,14^.

The combined effects in parents and their offspring of genetic factors, shared environmental exposures and lifestyle, in itself being determined by genetic and environmental factors, make it challenging to distinguish and quantify the impact of these factors^15^, but the rapidly expanding series of genetic variants associated with BMI improves the opportunities to disentangle the effects. A recent meta-analysis of genome-wide association studies (GWAS) in adults identified 941 BMI-associated common genetic variants explaining ~6% of BMI variance^16^, which was double the proportion of the variation explained by previous GWAS identified variants^17,18^. Building genetic risk scores (GRSs) by summation over this large number of genetic variants can provide strong genetic probes^19^, especially in settings, e.g. within families, where a the major part of the contribution to the variance in BMI from differences between families is controlled for. However, utilizing such GRSs to investigate the maternal and paternal genetic effects and the specific maternal effects is blurred by the transmission of only a random half of the parental alleles. By inferring parental transmissions of BMI-associated genetic variants from genetic information of the mother and her child by haplotype analysis we can construct GRSs of both parents’ transmitted and of the maternal non-transmitted alleles. In this study, the paternal transmitted alleles are those found in the child and not identified as maternally transmitted alleles. The maternal non-transmitted alleles are a genetic probe of specific maternal effects^20,21^.

We explored and quantified the influence of 941 parental transmitted and non-transmitted common BMI-associated variants, summarized in the GRSs, on children’s overweight in the large Danish National Birth Cohort of ~100,000 children. We employed a general population-based study design, where we compared children with overweight (CH-OW) and children of mothers with overweight (MO-OW) to a reference group of children of randomly selected mothers from the same population (REF) (a case-cohort design and an exposure-based cohort design, respectively). This study design allowed us to assess to what extent the parental transmitted GRSs contribute to child overweight and to the parent-child associations of overweight, whether maternal non-transmitted variants are associated with child overweight, and to explore if maternal overweight modifies the maternal and paternal genetic contributions.

## Results

**Figure 1:**
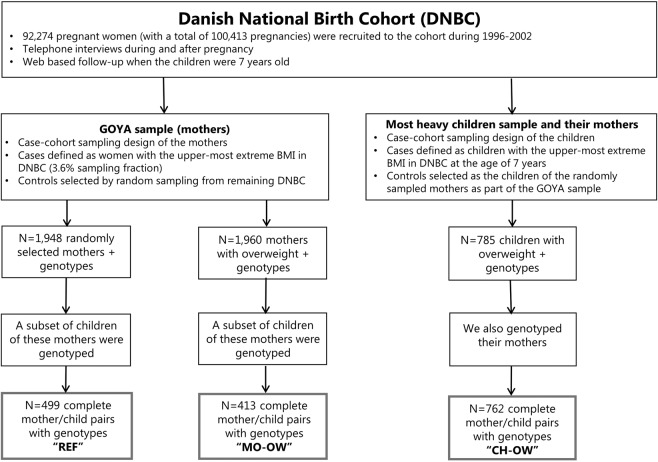
Participant flowchart and selection of mother-child pairs of the REF, MO-OW and CH-OW groups. CH-OW: Children with overweight and their mothers (children with overweight group); OW-MO: Mothers with overweight and their children (mothers with overweight group); REF: Randomly selected mothers and their children (reference group).

Figure 1 shows the participant flowchart and the selection of mother-child pairs that are part of the REF, MO-OW and CH-OW groups as part of the Danish National Birth Cohort, in which the child BMI was assessed at around age 7 years [median (interquartile range): 7.1 (7.0; 7.2) years].

### Phenotypic and GRS differences between the three sampling groups

Table 1 shows the key characteristics and differences in BMI and BMI expressed in standard deviation units (SD-units) between the three groups of mother-child pairs. As per sampling design, mothers of the MO-OW and children of the CH-OW groups had higher BMI and a higher proportion of overweight than mothers and children in the REF group. Mothers and fathers of the CH-OW groups had higher BMI and a higher proportion of overweight than mothers and fathers of the REF group, respectively. Fathers in the MO-OW had higher BMI and proportions of overweight than in the REF group, indicating some degree of phenotypic assortative mating.

**Table 1:** Study characteristics of the three groups of mother-child pairs within the Danish National Birth Cohort.

	REF		MO-OW		P for difference between the MO-OW and REF groups	CH-OW		P for difference between the CH-OW and REF groups
	N	Mean (SD) or %	N	Mean (SD) or %		N	Mean (SD) or %	
**BMI characteristics**								
Child BMI at 7 years (kg/m^2^)	496	15.6 (1.6)	412	16.8 (2.3)	<0.001	762	20.1 (2.0)	<0.001
Child BMI at 7 years (SD-units)		0.0 (1.00)		0.6 (1.1)			2.1 (0.5)	
Child overweight at 7 years (%)								
*No*		91.2		74.8	<0.001		0.0	<0.001
*Yes*		8.8		25.2			100.0	
Maternal BMI pre-pregnancy (kg/m^2^)	498	23.1 (3.2)	413	37.1 (3.3)	<0.001	762	25.1 (3.7)	<0.001
Maternal BMI pre-pregnancy (SD-units)		−0.1 (0.8)		3.2 (0.8)	<0.001		0.4 (0.9)	<0.001
Maternal overweight pre-pregnancy (%)								
*No*		76.1		0.0	<0.001		46.6	<0.001
*Yes*		23.9		100.0			53.4	
Paternal BMI (kg/m^2^)	395	25.1 (3.0)	335	26.8 (4.5)	<0.001	593	26.7 (3.7)	<0.001
Paternal BMI (SD-units)		−0.0 (0.9)		0.5 (1.4)	<0.001		0.5 (1.1)	<0.001
Paternal overweight (%)								
*No*		56.1		39.7	<0.001		35.2	<0.001
*Yes*		43.9		60.3			64.8	
**Other characteristics**								
Child gender (%)	499		413			762		
*Female*		48.9		48.9	0.99		49.6	0.85
*Male*		51.1		51.1			50.4	
Child age (years)	499	7.0 (0.3)	413	7.0 (0.3)	0.92	762	7.1 (0.3)	0.50
Maternal age (years)	499	30.5 (4.1)	413	30.1 (3.9)	0.10	762	30.6 (4.2)	0.81

We tested for differences in continuous characteristics between the mother-child groups at the extremities of the BMI distribution (MO-OW and CH-OW groups) and the REF group using t-tests and for differences in binary characteristics using two-proportions z-test integrated into the “prop.test” function in R software.

CH-OW: Children with overweight and their mothers (children with overweight group); OW-MO: Mothers with overweight and their children (mothers with overweight group); REF: Randomly selected mothers and their children (reference group).

**Figure 2:**
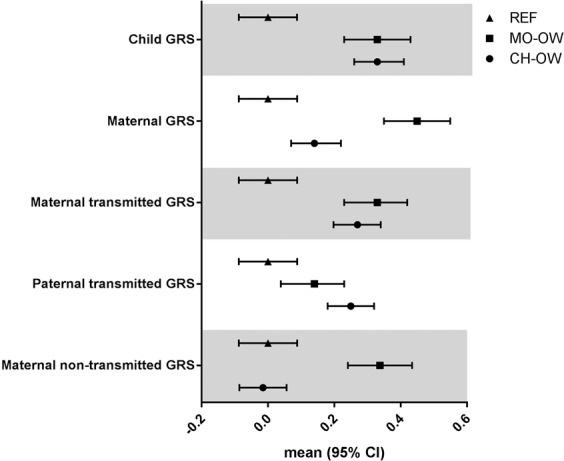
Overview of the mean (95% CI) of the various GRSs (expressed in SD-units) in each group of mother-child pairs. CH-OW: Children with overweight and their mothers (children with overweight group); GRS: Genetic risk score; OW-MO: Mothers with overweight and their children (mothers with overweight group); REF: Randomly selected mothers and their children (reference group).

The distributions of the 941 BMI-increasing risk alleles, summarized in the GRSs that were generated based on maternal transmitted and non-transmitted and paternal transmitted haplotypes are shown by numbers in Table 2 and as GRS in SD-units in Fig. 2. In spite of the differences in BMI [on average 1.5 SD-units higher BMI] and in overweight (100% versus 25%) (Table 1), children of the MO-OW and CH-OW groups carried the same number of BMI-increasing risk alleles, but a higher number than the children of the REF group. Mothers of the MO-OW carried a higher number of BMI-increasing risk alleles than the mothers of the REF group and the mothers of the CH-OW group. While the maternal transmitted GRS was higher in the MO-OW and CH-OW groups than in the REF group, there were no differences between the MO-OW and CH-OW groups. The MO-OW group had a higher maternal non-transmitted GRS than the REF group and the CH-OW group, but there was no difference between the CH-OW and REF group. Paternal transmitted GRS in the MO-OW group was slightly higher than in the REF group, indicating some genotypic assortative mating.

**Table 2:** Various GRSs comprised of 941 BMI-associated genetic variants of the three groups of mother-child pairs.

Genetic risk scores (GRSs) comprised of 941 BMI-associated genetic variants	REF		MO-OW		P for difference between the MO-OW and the REF groups	CH-OW		P for difference between the CH-OW and the REF groups
	N	Mean (SD)	N	Mean (SD)		N	Mean (SD)	
Child GRS								
*Number of BMI-increasing risk alleles*	499	901.9 (18.2)	413	908.0 (18.1)	<0.001	762	908.0 (18.3)	<0.001
Maternal GRS								
*Number of BMI-increasing risk alleles*	499	902.7 (17.4)	413	910.6 (18.1)	<0.001	762	905.3 (18.0)	0.01
Maternal transmitted GRS								
*Number of BMI-increasing risk alleles*	499	444.5 (12.2)	413	448.5 (12.1)	<0.001	762	447.8 (12.4)	<0.001
Paternal transmitted GRS								
*Number of BMI-increasing risk alleles*	499	446.3 (12.8)	413	448.0 (12.8)	0.04	762	449.5 (12.2)	<0.001
Maternal non-transmitted GRS								
*Number of BMI-increasing risk alleles*	499	446.8 (12.5)	413	451.0 (12.5)	<0.001	762	446.6 (12.5)	0.79

The genetic risk scores (GRSs) were comprised of 941 BMI-associated genetic variants, meaning that each mother and child carried 1882 alleles that were either BMI-increasing or decreasing. The GRSs are expressed as the number of the BMI-increasing alleles.

We tested for differences in GRSs comprised of 941 BMI-associated genetic variants between the mother-child groups at the extremities of the BMI distribution (MO-OW and CH-OW groups) and the REF group using t-tests.

CH-OW: Children with overweight and their mothers (children with overweight group); GRS: Genetic risk score; OW-MO: Mothers with overweight and their children (mothers with overweight group); REF: Randomly selected mothers and their children (reference group).

### Associations between parental BMI and various GRSs on continuous child BMI

Although the focus of this study is on child overweight, we assessed the GRSs on continuous child BMI within the REF group in order to compare our results with previous similar studies^13,14^. We analyzed SD-units for BMI and GRS unless otherwise stated. The child GRS was approximately normally distributed and associated with BMI of the child [0.19 SD (95% CI 0.11; 0.27), Table 3], explaining 3.9% of the variation in child BMI. Maternal and paternal BMI were associated with child BMI [maternal-child BMI association: 0.28 SD (95% CI 0.17; 0.39), paternal-child BMI association: 0.18 SD (95% CI 0.08; 0.28), Table 3]. The maternal and paternal transmitted GRSs were associated with child BMI [maternal transmitted GRS: 0.21 SD (95% CI 0.13; 0.30); paternal transmitted GRS: 0.10 SD (95% CI 0.02; 0.19), Table 3], while the maternal non-transmitted GRS was not associated with child BMI [0.04 SD (95% CI −0.05; 0.12), Table 3]. Notably, the ratio between the effect sizes of the maternal transmitted GRS and maternal BMI on child BMI (0.21 versus 0.28) was 0.75, while this ratio was 0.56 between the effect sizes of the paternal transmitted GRS and paternal BMI on child BMI (0.10 versus 0.18). However, the phenotypic associations were virtually unchanged after controlling for the respective genetically transmitted GRS [maternal-child BMI association adjusted for maternal transmitted GRS: 0.25 SD (95% CI 0.15; 0.36), paternal-child BMI association adjusted for paternal transmitted GRS: 0.18 SD (95% CI 0.08; 0.28)].

**Table 3:** Associations between parental BMI and the various GRSs with child BMI at 7 years of age in the reference group (REF).

Determining variable	N	β (95% CI)	P
Maternal BMI	494	0.28 (0.17; 0.39)	5.1E-07
Paternal BMI	392	0.18 (0.08; 0.28)	0.0003
Child GRS	495	0.19 (0.11; 0.27)	9.2E-06
Maternal GRS	495	0.14 (0.06; 0.22)	0.001
Maternal transmitted GRS	495	0.21 (0.13; 0.30)	3.8E-07
Paternal transmitted GRS	495	0.10 (0.02; 0.19)	0.01
Maternal non-transmitted GRS	495	0.04 (−0.05; 0.12)	0.38

Results from multiple linear regression analyses are given as β in SD-units (95% CI) highlighting the effect of parental BMI (SD-units) and the various GRSs (SD-units) on child BMI). Sample size (N) for respective analysis is reported. The applied regression formulas in R were lm(child BMI ~ determining variable).

GRS: Genetic risk score.

### The associations of phenotypic and GRS on the odds of child overweight

#### Case-cohort design analysis of the CH-OW and REF groups

Using a case-cohort design with the genotyped mother-child pairs of the CH-OW group and the REF group, we assessed the contribution of the phenotypic and GRS differences on the odds of child overweight (= odds of the child being selected into the CH-OW group). Table 4 indicates that maternal BMI and paternal BMI had a strong association with childhood overweight [Odds ratio (OR): 2.01 (95% confidence interval (CI) 1.74; 2.34) and 1.64 (95% CI 1.43; 1.89) per SD-unit for paternal BMI, respectively]. The maternal and paternal transmitted GRSs were associated with increased odds for childhood overweight at the same magnitude [OR: 1.30 (95% CI 1.16; 1.46) and 1.30 (95% CI 1.16; 1.47) per SD-unit, respectively]. The ratio between the excess odds ratio of the maternal transmitted GRS and maternal BMI on childhood overweight (0.30 versus 1.01) was 0.30, while this ratio was 0.47 between the paternal effect sizes (0.30 versus 0.64). However, adjustment of the phenotypic associations of the maternal and paternal BMI with childhood overweight for the maternal and paternal transmitted GRSs, respectively, did not change the results materially [OR: 1.95 (95% CI 1.68; 2.28) and 1.63 (95% CI 1.42; 1.88) per SD-unit, respectively]. The maternal non-transmitted GRS was not associated with childhood overweight [OR: 0.98 (95% CI 0.88; 1.10) per SD-unit]. The association between maternal GRS and childhood overweight was as expected approximately half of the excess OR of the maternal transmitted GRS (0.15 versus 0.30). The child’s own GRS was also associated with increased odds of childhood overweight [OR: 1.39 (95% CI 1.24; 1.57) per SD-unit].

**Table 4:** Results of the case-cohort design based analysis to estimate the associations between parental BMI and the various BMI-increasing GRSs and childhood overweight.

Determining variable	N	OR (95% CI)	P
Maternal BMI	1260	2.01 (1.74; 2.34)	4.2E-20
Paternal BMI	987	1.64 (1.43; 1.89)	2.5E-12
Child GRS	1261	1.39 (1.24; 1.57)	1.6E-08
Maternal GRS	1261	1.15 (1.02; 1.29)	0.01
Maternal transmitted GRS	1261	1.30 (1.16; 1.46)	4.5E-06
Paternal transmitted GRS	1261	1.30 (1.16; 1.47)	1.0E-05
Maternal non-transmitted GRS	1261	0.98 (0.88; 1.10)	0.79

Results from logistic regression analyses are given as OR (95% CI) from crude analysis showing the effect of parental BMI (SD-units) and the various GRSs (SD-units) on childhood overweight. In this case-cohort design based analysis, we pooled the CH-OW and REF groups to estimate the odds of childhood overweight (= odds for being selected into the CH-OW group), and the applied regression formulas in R were glm(indicator variable for being selected into the REF or CH-OW group (0/1) ~ determining variable).

CH-OW: Children with overweight and their mothers (children with overweight group); GRS: Genetic risk score; OW-MO: Mothers with overweight and their children (mothers with overweight group); REF: Randomly selected mothers and their children (reference group).

#### Exposure-based cohort design analysis of the MO-OW and REF groups

Using an exposure-based cohort design of the MO-OW and REF groups adjusted for binary indicator of these groups, we confirmed the overall patterns of the above estimated associations of the various phenotypic and GRS variables on the odds for childhood overweight (Table 5). We found that both, maternal and paternal BMI were associated with increased odds for childhood overweight [OR: 1.43 (95% CI 1.14; 1.78) and 1.38 (95% CI 1.18; 1.61) per SD-unit, respectively]. The maternal and paternal transmitted GRSs were both associated with increased odds for childhood overweight [OR: 1.37 (95% 1.14; 1.65) and 1.39 (1.16; 1.68) per SD-unit, respectively], while the maternal non-transmitted GRS was not associated with childhood overweight [OR: 0.98 (95% CI 0.82; 1.17) per SD-unit]. The odds for child overweight associated with maternal overweight [OR: 3.48 (95% CI 2.39; 5.14) per SD-unit] dropped only little by adjustment for the maternal transmitted GRS [to OR: 3.19 (95% CI 2.18; 4.73) per SD-unit], and also the associations with parental BMI changed only little when adjusted for the parental transmitted GRS [maternal OR: 1.39 (95% CI 1.11; 1.74) and paternal OR 1.35 (95% CI 1.16; 1.59) per SD-unit]. The child’s own GRS was associated with increased odds of childhood overweight, and the excess OR of the maternal GRS on childhood overweight was, as expected, approximately half of the OR of the maternal transmitted GRS (0.18 versus 0.37).

**Table 5:** Results of the exposure-based design analysis to estimate the associations between parental BMI and the various BMI-increasing GRSs and childhood overweight.

Determining variable	N	Adjusted models		Interaction models		
		OR (95% CI)	P - value	REF, OR (95% CI)	MO-OW, OR (95% CI)	P (interaction)
Maternal BMI	911	1.43 (1.14; 1.78)	0.002	2.04 (1.40; 2.95)	1.18 (0.89; 1.55)	0.02
Paternal BMI	730	1.38 (1.18; 1.61)	5.1E-05	1.66 (1.17; 2.35)	1.31 (1.11; 1.56)	0.24
Child GRS	912	1.58 (1.30; 1.92)	3.6E-06	2.30 (1.63; 3.32)	1.31 (1.05; 1.66)	0.009
Maternal GRS	912	1.18 (0.99; 1.41)	0.07	1.70 (1.24; 2.36)	0.99 (0.80; 1.23)	0.006
Maternal transmitted GRS	912	1.37 (1.14; 1.65)	8.7E-04	2.53 (1.78; 3.70)	1.04 (0.83; 1.31)	5.1E-05
Paternal transmitted GRS	912	1.39 (1.16; 1.68)	5.0E-04	1.65 (1.20; 2.29)	1.28 (1.02; 1.61)	0.21
Maternal non-transmitted GRS	912	0.98 (0.82; 1.17)	0.82	1.21 (0.89; 1.66)	0.88 (0.70; 1.10)	0.10

Results from logistic regression analyses are given as OR (95% CI) showing the effect of parental BMI (SD-units) and the various GRSs (SD-units) on childhood overweight. In this exposure-based analysis, we pooled the MO-OW and REF groups to estimate the odds of childhood overweight (= child with overweight at 7 years). The adjusted logistic regression models were adjusted for group indicator (REF group/MO-OW group). We also conducted interaction analyses to estimate whether the presence of maternal overweight (= mother selected into the MO-OW group) is modifying the influence of the various determining variables on the odds of childhood overweight. The unadjusted OR (95% CI) for the group indicator is 3.48 (2.39; 5.14). The applied regression formulas in R were glm(child overweight status (0/1) ~ determining variable + group indicator variable) for the adjusted models, and glm(child overweight status (0/1) ~ determining variable + group indicator variable + group indicator variable*determining variable) for the interaction models.

GRS: Genetic risk score; MO-OW: Mothers with overweight and their children (mothers with overweight group); REF: Randomly selected mothers and their children (reference group).

We further used the sampling of the exposure-based cohort design to investigate whether the presence of maternal overweight in the MO-OW group relative to the REF group modified the influence of the determining variables on the odds of childhood overweight. We found that the association of maternal BMI, GRS, and transmitted GRS as well as child GRS with childhood overweight was stronger among the REF group than among the MO-OW group, whereas the associations with paternal BMI and transmitted GRS were less clear, indicating that maternal overweight may diminish these effects (Table 5).

## Discussion

The present study explored contributions of the most recent GWAS-based panel of 941 common genetic variants associated with adult BMI to the understanding of the parent-child associations in adiposity. By haplotype analysis and summation of the variants in GRSs of the mothers and their children, the associations with maternal and paternal transmitted, as well as maternal non-transmitted genetic variants - all computed on the same genotypes - were disentangled. The results highlight the effects of the currently identifiable common genetic contributions by parental transmitted GRSs to childhood overweight and showed that little of the associations of maternal and paternal BMI with child overweight may be explained by these effects. There was little evidence of specific maternal effects – probed by the maternal non-transmitted GRS – on child BMI. Furthermore, our results suggest that maternal overweight may diminish the effects of the maternal transmitted GRS on overweight during childhood.

A major strength of the present investigation is the unique study design including mother-child pairs with extreme BMI conditions within the large Danish National Birth Cohort, namely mothers with overweight and their children (MO-OW group), children with overweight and their mothers (CH-OW group) and a reference group of randomly selected mothers and their children (REF). With regard to the specific aims of the study, the statistical power of this case-cohort and an exposure-based cohort design is demonstrated by the precision of the estimates. The transmitted and the non-transmitted maternal GRSs allow us to distinguish between associations of genetic nature (association with maternal transmitted GRS) and associations of specific maternal effects before, during or after the pregnancy (association with maternal non-transmitted GRS, also genetic nurturing effects, here assessable only on the maternal side). Using information on genome-wide genotypes allowed us to check and confirm the true genetic relatedness between each mother and her child in the included mother-child pairs, excluding bias due to non-maternity and non-paternity (since the paternal transmitted alleles were complimentary approximated on the basis of the genetic information of the mother and her child and thus represents the “true biological” father).

On the other hand, various limitations of our study must be considered in the interpretation of the results. Concerning generalizability of the outcomes of our analyses, we acknowledge that our analyses were conducted in a Western population with a lower prevalence of childhood obesity than most other countries in this area^22^. The mothers enrolled in the Danish National Birth Cohort had on average a higher educational level and were on average of somewhat higher social class than the general population^23^, which may have weakened the effects of the transmission of the genetic predisposition^24^. Although information on current height and weight based on self-report has a high accuracy and reliability, there may be biases, especially in the extremes^25^. Information based on reporting height and weight of others is less accurate and less reliable^26^, so there may have been greater random and possibly also systematic errors in the reporting of child and paternal height and weight than for the mothers. Moreover, the mothers reported on the fathers’ height and weight when the child was around 18 months old, adding uncertainty due to possible postpartum influences. Although the probability of non-paternity within the Danish National Birth Cohort appears very low^2^, we cannot exclude that it may have biased the results of the paternal phenotypic analyses. The uncertainties about the paternal relative to maternal anthropometrics imply that the results involving the paternal phenotypes will tend to be weaker, and the deviations from maternal results may be methodological artefacts rather than parent-of-origin effects. In contrast to the strengths of the between-group comparisons, the limited sample size of each selected group of mother-child pairs for genotyping provides little power to detect associations within each group, which affects our results of analyses of the REF group alone and of the interaction analyses of the REF versus the MO-OW group. Furthermore, the results from analyses within the groups of phenotypic extremes should be cautiously interpreted due to possible biases induced both by the BMI-based selection of children and mothers and by the influences on the observed associations by the phenotypic and genotypic assortative mating in mothers with overweight (MO-OW group). Assortative mating between spouses according to BMI has been shown in previous studies^27^, and may allow for phenotypic expression of alleles with recessive and epistatic effects. In this study we only had genotype data on mothers and children providing information of paternal transmitted GRS only from the child alleles not assigned to transmission from the mother. In determining the transmitted and non-transmitted alleles without access to paternal genotype data there is a bias in determining allelic transmission, meaning that for lower frequency SNPs the paternal minor allele is more likely to be determined than the maternal minor allele. We constructed the various GRSs assuming additive effects of the alleles and did not account for possible gene-gene interactions in our analyses. Even though the GRSs used in this study were generated from GWAS of adult BMI^16^ and showed a strong association with child BMI and overweight, the discovery of genetic variants specifically associated with childhood BMI and overweight^28,29^, implies that the current results may underestimate the contribution from transmitted genetic variants to the child BMI and overweight.

The genetic susceptibility to BMI is heterogeneous and both common and rare genetic variants contribute^30^. For the majority of the population, overweight is polygenic and multiple common genetic variants with small effects contribute to its susceptibility^30^. The 941 common genetic variants included in the GRS calculations explain ~6% of the overall BMI variance in the adult population^16^. While these variants make a stronger genetic probe, compared to a single *FTO* variant (explaining ~0.3% of BMI variance)^17^ or GRS based on 97 genetic variants (explaining ~3% of BMI variance)^18^ that were utilized as genetic instruments in previous studies^31,32^, they still explain only a small fraction of the heritability as estimated from family-based phenotype studies. Recently, methods have been developed that allow estimating heritability estimates more robustly, producing estimates comparable to the family-based studies, with heritability for BMI at 30–40%^33–36^. It is expected that the missing heritability of BMI with the available 941 variants is accounted for by common variants that did not reach genome-wide significance in available GWAS of BMI and rare variants that are expected to be discovered in the near future with improved imputation panels, very large discovery cohorts and whole-genome sequencing approaches^33,36^. On the other hand, in studies of the within-family associations, the GRS will be a stronger genetic probe than indicated by the level of explained variance in the general population because of the control of variance due to between-family differences. This may be the reason for the relatively high ORs for the parental transmitted GRSs compared with the ORs for the parental BMI. However, in spite of these ORs, adjustment for the respective transmitted GRS when estimating the association of parental BMI with child BMI and child overweight had minimal effects, corresponding to the combination of high degree of unexplained genetic variance by the GRSs and the effects of shared environmental factors.

We found in the REF group that both maternal and paternal transmitted GRSs were associated with childhood BMI, while the maternal non-transmitted GRS was not. These results are compatible with the findings from the two studies on child adiposity in the Avon Longitudinal Study of Parents and Children that applied intergenerational Mendelian Randomization analyses using maternal genotypes adjusted for child genotypes as instrumental variables for specific effects of maternal BMI on child adiposity^13,31^. The study by Lawlor *et al*.^31^ included a sample of 3,263 children aged 9–11 years where information on fat mass was assessed by dual energy X-ray absorptiometry. The use of maternal *FTO* genotype (with control for child *FTO* genotype) as instrumental variable for maternal BMI did not show an association with later offspring adiposity, suggesting that there was no strong evidence for a specific causal effect of greater maternal BMI. The second study by Richmond *et al*.^13^ included 3,720 children aged 7 years of the same cohort and a replication sample of 2,337 children aged 6 years of the Dutch Generation R study. GRSs in these samples were calculated based on 97 and 32 BMI-associated genetic variants, respectively, and the instrumental variable in the intergenerational Mendelian randomization analysis was maternal GRS adjusted for offspring GRS. The results of this main analysis showed little evidence of an important causal effect of greater maternal BMI on later offspring adiposity. In sensitivity analysis, Richmond *et al*.^13^ also estimated maternal transmitted and non-transmitted GRSs and found that the maternal non-transmitted haplotype based GRS was not associated with child BMI, while the maternal transmitted haplotype based GRS was associated with child BMI. Taken together, these findings support the validity of our results in the REF group, and they are in line with our findings on child overweight. Similarly, the results of a recent meta-analysis suggested limited evidence that maternal and paternal non-transmitted alleles affect offspring adult BMI through their impacts on the parents and other relatives, also known as genetic nurturing^37^.

Maternal overweight seems to attenuate the effect of the maternal transmitted BMI-increasing BMI GRS on childhood overweight. While we acknowledge the particular limitations in this part of the study, we find the results worth interpreting. Thus, the dilution of the associations in the maternal overweight group may to some extent be a statistical artefact, having been exaggerated by the truncation of the maternal BMI distribution and the different prevalence of overweight in the REF and MO-OW group among mothers, fathers and children. However, assuming that there is also a true interaction behind these results, which would need testing in an independent study, we speculate that the selected overweight mothers had reached their overweight status for various genetic and/or environmental reasons that also influenced the risk of overweight in the children, but were not probed by the transmitted or the non-transmitted GRSs. This possibility fits with a bivariate analysis of twins indicating that the magnitude of twin correlation is smaller (i.e. the discordance between the twin pairs was greater) in the part of the overweight range of the distribution of the twin pairs than in those with normal weight^38,39^.

In conclusion, we observed that both maternal and paternal transmissions of adult BMI-associated risk alleles contributed to increased odds of childhood overweight. Moreover, there was no evidence of specific effects of greater maternal BMI on childhood overweight as probed by the non-transmitted GRS. Even though the 941 genetic variants explain only a small proportion of BMI variation in the general population, we demonstrate that they contribute to the parent-child associations in overweight. If we assume that the maternal non-transmitted GRS is an adequate probe of specific effects of maternal adiposity acting on the child’s risk of overweight, then our findings do not support such an effect. Maternal overweight may to some extend attenuate the effect of the maternal transmitted GRS on childhood BMI, possibly because of dilution of the effects by other genetic or environmental factors. The results of our study encourages continued search for the genetic and/or environmental factors influencing child BMI and overweight beyond the currently known genetic variants constituting the GRS based on adult BMI.

## Methods

### Cohort and selection of participants

We used data from selected mother-child pairs who participated in the Danish National Birth Cohort. This is a population-based prospective birth cohort study and enrolled a total of 100,413 pregnancies among 92,274 women from all over Denmark in 1996–2002^40^. The study website contains information about the available data: http://www.dnbc.dk/data-available. As illustrated in Fig. 1, we included three selected groups of mother-child pairs with complete information on BMI and genotype for both the mother and her child (at around age 7 years) into the present analysis: randomly selected mothers and their children (reference group, REF, n = 499 mother-child pairs); mothers with overweight and their children (mothers with overweight group, MO-OW, n = 413 mother-child pairs); overweight children with the highest BMI at 7 years of age and their mothers (children with overweight group, CH-OW, n = 762 mother-child pairs). Only singleton births were included in this analysis because of markedly different intrauterine growth patterns between singleton and multiple births. Each mother gave written informed consent at enrolment into the study. The genotyping of mother-child pairs was approved by the Danish Ethical Committee (1-10-72-195-13 and 1-10-72-261-14). The study was conducted in accordance with the principles of the Declaration of Helsinki.

### Assessing parental and offspring body mass index

Self-reported pre-pregnancy weight and height were obtained for the mothers during the telephone interview in gestational week 16. During the 18 months postpartum interview, the mothers gave information on height and weight of the father. A web-based follow-up was conducted when the children were at around 7 years old, and included information on the child’s weight and height. Weight and height data in the 7-year follow-up was validated against measured height and weight from school health records in a small sub-sample of the Danish National Birth Cohort^41^. BMI of parents and children was calculated by weight/height^2^ (kg/m^2^). For the present study, internally standardised basic z-scores for parental (maternal and paternal) BMIs, as well as sex- and age-specific z-scores for the child BMI (in month categories; by the Lambda-Mu-Sigma procedure^42,43^), were derived and presented as SD-units.

### Genotyping, SNP selection and GRS construction

Blood samples were collected from the mothers twice during pregnancy and cord blood was collected at birth. Mothers of the REF and MO-OW groups were genotyped using the Illumina Human610-Quad Beadchip; the quality control steps for exclusion of SNPs and individuals have been previously described in detail^44^. The remaining mothers and children (mothers of the CH-OW and children of all three groups) were genotyped using the Illumina Infinium HumanCoreExome Beadchip (Illumina, San Diego, CA, USA) and genotypes were called using the Genotyping module, version 1.9.4 of GenomeStudio software, version 2011.1 (Illumina, San Diego, CA, USA). During quality control, we excluded closely related individuals and samples with extreme inbreeding coefficients, mislabeled gender, mislabeled mother-child pairs that we found were not genetically related, call rate <95%, duplicates and individuals identified as ethnic outliers. We applied a >95% genotype call rate filter for the inclusion of SNPs. Genotype imputations were conducted using the Haplotype Reference Consortium (HRC, release 1) at the server at the Sanger institute for all mothers and children included in the present analysis^45^.

For each mother and child, we extracted 941 genetic variants robustly associated with BMI from the HRC imputed genotype dataset. The 941 genetic variants were associated with BMI at a revised genome-wide significance threshold (P < 1×10^−8^) in the large meta-analysis by Yengo *et al*. of genome-wide association studies for adult BMI including ~700,000 individuals of European ancestry^16^. We used the 941 near-independent genetic variants to generate weighted BMI-increasing GRSs by summing the genotype dosages of the BMI-increasing alleles weighted by the effect sizes of the variants. For descriptive information, the scores were then rescaled to reflect the number of BMI-increasing alleles carried by an individual using a previously described method^46^. We standardized all GRSs into z-scores and present these as SD-units.

### Generating maternal transmitted and non-transmitted and paternal transmitted haplotype based GRS

We derived parental allelic transmission for all genotyped 1,674 mother-child pairs using a previously described method^20^. First, maternal and child genotypes were converted into best guess genotypes. Where either the mother or her child, or both were homozygous, allelic transmission was unambiguous to determine. Where both mother and offspring were heterozygous for the SNP of interest, we used phased imputation generated using SHAPEIT2 to examine the haplotypes in the region of the genetic variant of interest to estimate allelic transmission. We then generated maternal transmitted and non-transmitted and paternal transmitted haplotype based GRSs based on the 941 BMI-associated genetic variants in Yengo *et al*.^16^, weighted by effect-size, and then rescaled and standardized as outlined above. The code that we used to infer maternally and paternally inherited alleles from mother-child is available on GitHub https://github.com/rnbeaumont/poe_generator. The validity of the GRSs were verified by the findings that the correlations of maternal total and transmitted GRSs and paternal transmitted GRS with child GRS and the correlations of maternal total GRS with transmitted and non-transmitted GRSs ranged between 0.44 and 0.59 (with the expectations being around 0.50), whereas the correlation of the maternal non-transmitted GRS with the child GRS ranged between −0.06 and 0.15 (with the expectation of it being around 0.0).

### Statistical analyses

All analyses were conducted using R, version 3.3.1. None of our models was adjusted for age or sex of the child because, by construction, child BMI z-score is age- and sex-specific. Unless otherwise stated, SD-units for BMI and GRS were analysed.

We conducted within-group analysis of the REF group with continuous child BMI as the outcome. The associations between parental BMI and child BMI were analysed by multiple linear regression and we investigated whether the observed associations between parental BMI and child BMI would persist after additional adjustment of the regression model for the respective maternal and paternal transmitted haplotype based GRS. The associations between the various GRSs and child BMI were also analysed by multiple linear regression.

In the case-cohort analysis, we combined the CH-OW and REF groups and conducted logistic regression analyses to estimate the odds of childhood overweight (= the odds of being selected into the CH-OW group), derived as OR with 95% CI. In the exposure-based cohort design analysis, we combined the MO-OW and REF groups and conducted logistic regression analyses, adjusted for group indicator (REF/MO-OW group), to estimate the odds of childhood overweight at age 7 (disregarding the possible interactions with the group indicator). We conducted analyses estimating the association of parental BMI with childhood overweight when adjusted for the respective maternal and paternal transmitted GRS. Interaction analyses were conducted to test whether the presence of maternal overweight (= mother selected into the MO-OW group) is modifying the influence of the tested exposure variables (maternal BMI, paternal BMI, child GRS, maternal GRS, maternal transmitted GRS, paternal transmitted GRS and maternal non-transmitted GRS) on the odds of childhood overweight (p-interaction).

## Data Availability

Relevant data for the present study are within the paper and its Supporting Information files. Access to additional individual data underlying the findings may be approved with some restrictions. Data is available from the Danish National Birth Cohort and can be requested through the steering committee of the study who can be contacted under dnbc-research@ssi.dk. More information regarding access to data can be found on the Danish National Birth Cohort website http://www.dnbc.dk/access-to-dnbc-data.
